# Association between the HALP score and survival of patients with esophageal or gastro-esophageal junction cancer: a meta-analysis

**DOI:** 10.3389/fonc.2026.1831724

**Published:** 2026-06-23

**Authors:** Wu Wang, Junhai Chen, Tianbao Yang

**Affiliations:** Department of Cardiothoracic Surgery, The Affiliated Hospital of Putian University, Putian, China

**Keywords:** esophageal cancer, gastro-esophageal junction cancer, inflammation, nutrition, survival

## Abstract

**Background:**

The hemoglobin, albumin, lymphocyte, and platelet (HALP) score is a composite biomarker that reflects the nutritional and inflammatory status. However, the prognostic value of baseline HALP scores in patients with esophageal cancer (EC) or gastro-esophageal junction cancer (GEC) remains incompletely defined.

**Aim:**

To systematically evaluate the association between baseline HALP scores and survival outcomes in EC or GEC through a meta-analysis.

**Methods:**

PubMed, Embase, and Web of Science were searched for longitudinal observational studies that evaluated the baseline HALP score, and overall survival (OS) and/or progression-free survival (PFS) of EC or GEC patients. Then, the hazard ratios (HRs) with 95% confidence intervals (CIs) used to compare the low *vs.* high HALP scores were pooled using random-effects models, incorporating the potential influence of heterogeneity.

**Results:**

Ten cohort studies that comprised of 1, 953 patients were included. Compared with patients with high HALP scores, patients with low HALP scores had significantly poorer OS (HR: 1.74, 95% CI: 1.53-1.98, *I^2^* = 0%). This association was consistent across subgroups defined by histology, age, sex proportion, treatment modality, HALP score cut-off values, follow-up duration, and analytic models. In five studies that reported the PFS, low HALP scores were associated with worse PFS (HR: 1.82, 95% CI: 1.40-2.37, *I^2^* = 0%). The sensitivity analyses confirmed the robustness of the findings.

**Conclusion:**

Low baseline HALP scores are independently associated with poorer OS and PFS in EC or GEC patients.

**Systematic review registration:**

PROSPERO, identifier CRD420261300969.

## Introduction

Esophageal cancer (EC) and gastro-esophageal junction cancer (GEC) are among the most aggressive gastrointestinal malignancies worldwide, with a substantial geographic variation in incidence and histological distribution (1, 2). EC remains as one of the leading causes of cancer-related mortality, particularly in East Asia, where esophageal squamous cell carcinoma (SCC) predominates, while adenocarcinoma is more common in Western countries, and frequently involves the gastro-esophageal junction (3, 4). Despite the advances in multimodal management, including curative-intent surgery, neoadjuvant or definitive chemoradiotherapy, perioperative chemotherapy, and the recent incorporation of immunotherapy (5), long-term outcomes remain poor, with 5-year survival rates generally below 30% for most patients (6). Furthermore, the prognosis is highly heterogeneous, even among patients with similar tumor stage and treatment strategies, reflecting the limitations of conventional staging systems in fully capturing host- and tumor-related biological differences (7). Accordingly, there is growing interest in identifying simple, reproducible, and clinically accessible biomarkers that can improve prognostic stratification, guide treatment decision-making, and optimize peri-treatment management in patients with EC or GEC (8).

The hemoglobin, albumin, lymphocyte, and platelet (HALP) score is a composite index calculated as hemoglobin (g/L) × albumin (g/L) × lymphocyte count (/L) ÷ platelet count (/L), integrating key components, including nutritional status, systemic inflammation, and immune competence (9). Originally proposed as a prognostic marker for gastrointestinal and other solid tumors, the HALP score has the advantage of being inexpensive, objective, and derived from routine laboratory parameters (10, 11). A low HALP score reflects anemia, hypoalbuminemia, lymphopenia, and thrombocytosis, which are conditions that are individually associated with impaired treatment tolerance, enhanced tumor progression, and unfavorable survival (12). Biologically, HALP may influence clinical outcomes in EC through the combined effects of malnutrition-related frailty, inflammation-driven tumor growth, and weakened antitumor immune surveillance (13, 14). In recent years, multiple observational studies have explored the prognostic value of the baseline HALP score in patients with EC or GEC, receiving various treatment modalities (15–24). However, the reported effect sizes and consistency of its association with survival outcomes have varied across studies, and no consensus has been reached (9). Therefore, a meta-analysis was conducted to systematically synthesize available longitudinal evidence, and quantitatively evaluate the association between the baseline HALP score, and overall survival (OS) and progression-free survival (PFS) in patients with EC or GEC, thereby clarifying its potential role as a prognostic biomarker in this high-risk population.

## Materials and methods

The present meta-analysis followed the PRISMA 2020 guidelines (25) and the Cochrane Handbook for Systematic Reviews and Meta-Analyses (26) for the protocol design, data extraction, statistical analysis, and results reporting. The study protocol was registered in PROSPERO under ID CRD420261300969.

### Literature search

Relevant studies for the present meta-analysis were identified through a comprehensive search in PubMed, Embase, and Web of Science, which are major international biomedical databases with broad coverage of oncology and prognostic studies. Since the present meta-analysis focused on observational prognostic studies, rather than interventional trials, these databases were considered sufficient to capture the relevant peer-reviewed evidence. A broad range of search terms was used, which included the following (1): “hemoglobin, albumin, lymphocyte, and platelet” OR “HALP” (2); “esophageal” OR “esophagus” OR “oesophageal” OR “oesophagus” OR “gastro-esophageal junction” OR “gastroesophageal junction”; and (3) “carcinoma” OR “adenocarcinoma” OR “cancer” OR “tumor” OR “malignancy” OR “malignant” OR “neoplasm”. The search was limited to human studies and full-length articles published in the English language in peer-reviewed journals. In addition, references from relevant original and review articles were manually screened for further eligible studies. The search spanned from database inception to May 24, 2026.

### Inclusion and exclusion criteria

The eligibility criteria for studies were established using the PICOS principle.

P (Population): Adult patients with EC or GEC, without limitations of cancer stage, histology, or main anticancer treatments;

I (Exposure): Participants with low HALP scores at baseline, with cut-offs for defining a low HALP score consistent with the value used in the original studies;

C (Comparison): Participants with high HALP scores at baseline;

O (Outcome): OS and/or PFS, compared between patients with high *vs.* low HALP scores at baseline. OS was generally defined as the interval from initiation of treatment to death from any cause, while PFS was defined as the time from treatment initiation to documented disease progression or death, whichever occurred first;

S (Study design): Observational studies with longitudinal follow-up, such as prospective or retrospective cohort studies, nested case-control studies, and the *post-hoc* analysis of randomized controlled trials (RCTs).

Studies were excluded when these were reviews, editorials, meta-analyses, preclinical studies, and studies that included pediatric patients, and patients with non-EC or non-GEC cancer, did not evaluate the HALP score as exposure, or did not report the outcome of interest. When multiple publications used overlapping cohorts, merely the study with the most complete data or the largest sample size was included.

### Study quality assessment and data extraction

Two authors independently conducted the literature search, study selection, quality assessment, and data extraction, resolving discrepancies through discussion with the corresponding author. The study quality was evaluated using the Newcastle-Ottawa scale (NOS) (27), which assesses the selection, confounding control, and outcome measurement. The scores ranged from 1 to 9, where 9 represented the highest quality. Studies with NOS scores of 7 or above were considered of high quality. The data extracted for analysis included the study characteristics (first author, year, study country, and design), patient characteristics (diagnosis, patient number, mean age, sex distribution, cancer stage, and main treatment), methods for determining the cut-off of the HALP score, cut-off values for the HALP score, median follow-up durations, outcomes reported, and variables adjusted when the associations between the HALP score and survival outcomes of patients with EC or GEC were estimated.

### Statistical analysis

The association between the HALP score and survival of patients with EC or GEC was summarized in hazard ratios (HRs) with the corresponding 95% confidence intervals (CIs). HRs and the standard errors were calculated from the 95% CIs or *p*-values, and log-transformed to stabilize the variance and normalize the distribution (26). In order to assess the heterogeneity, the Cochrane Q test and *I^2^* statistics were used (18), with *I^2^* < 25%, 25~75%, and >75% indicating mild, moderate, and substantial heterogeneity among the included studies, respectively. A random-effects model was used to synthesize the results, while accounting for variability across studies (26). The sensitivity analysis was performed by excluding one study at a time to evaluate the robustness of the findings (27). Additional sensitivity analyses were performed, excluding studies in which the cut-off values of the HALP score were not determined using receiver operating characteristic (ROC) curve analysis, in order to evaluate the potential influence of heterogeneity in the cut-off derivation methods on the pooled results. Furthermore, sensitivity analyses were performed, including only studies with NOS scores of ≥7, in order to evaluate the potential influence of relatively lower-quality studies on the pooled results. For the primary outcome of OS, predefined subgroup analyses were performed to evaluate the influence of the study characteristics on the results, which included the histology type of the cancer (SCC *vs.* adenocarcinoma or SCC), mean age of the patients, proportion of men, main treatment (surgical *vs.* non-surgical), cut-off values of the HALP score, follow-up duration, and analytical model (univariate *vs.* multivariate). Publication bias was assessed using funnel plots, visual asymmetry inspection, and Egger’s regression test (23). A *p-*value of <0.05 indicated statistical significance. The statistical analysis was conducted using the RevMan (Version 5.3; Cochrane Collaboration, Oxford, UK) and Stata (version 17.0; Stata Corporation, College Station, TX, USA) software.

## Results

### Study identification

**Figure 1:**
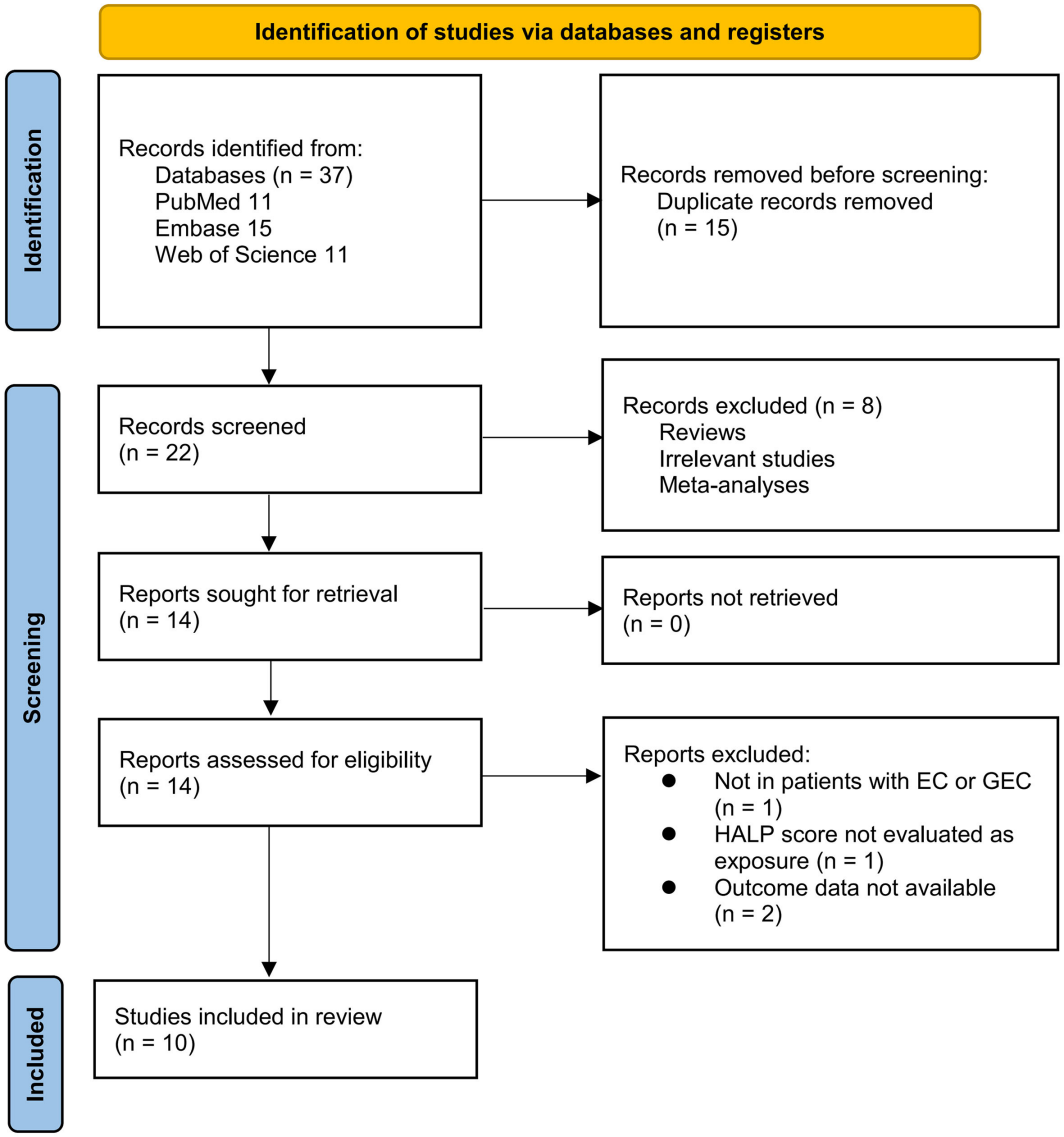
Flowchart for the database search and study inclusion.

**Figure 1** outlines the study selection process. Initially, 37 articles were identified across the three databases, with 15 duplicates removed. After title and abstract screening, eight articles were excluded for not meeting the meta-analysis criteria. Then, the full texts of the remaining 14 studies were independently reviewed by two authors, leading to the exclusion of four studies based on the reasons detailed in **Figure 1**. Ultimately, 10 studies were included for the quantitative analysis (15–24).

### Overview of the study characteristics

The characteristics of the included studies are summarized in **Table 1**. A total of 10 longitudinal observational studies published between 2017 and 2025 were included. These comprised of nine retrospective cohort studies (15–22, 24) and one prospective cohort study (23), which were conducted across China, Japan, Turkey, and Nepal. Overall, 1, 953 patients with EC or GEC were analyzed. Eight studies included patients with esophageal SCC only (15–17, 19–22, 24), and two studies included patients with esophageal or gastro-esophageal junction adenocarcinoma (18, 23). The mean age of the participants ranged from approximately 55.8 to 70.0 years old, and the proportion of men widely varied (42.2-100.0%). Furthermore, these patients covered a broad spectrum of disease stages (from resectable early-stage disease to locally advanced and unresectable disease), and received heterogeneous treatments, including curative esophagectomy, neoadjuvant chemotherapy or chemoradiotherapy, definitive chemoradiotherapy, and chemo-immunotherapy-based multimodal strategies. The baseline HALP score was consistently assessed prior to treatment initiation, with cut-off values mainly determined using the ROC curve (16–20, 23), median of HALP score (15), or previously reported thresholds (22). The cut-off values of the HALP score varied from 20.0 to 50.0. Furthermore, the median follow-up durations substantially varied across studies, which ranged from 1.0 to 52.8 months. For the outcome, OS was analyzed in nine studies (15, 16, 18–24), while PFS was reported in five studies (15, 19–21, 24). Furthermore, three studies (20, 22, 23) only provided unadjusted estimates, while the other seven studies (15–19, 21, 24) reported multivariable-adjusted outcome data.

**Table 1:** Characteristics of the included studies.

Study	Country	Design	Diagnosis	No. of patients	Mean age (years)	Men (%)	Cancer stage	Main treatment	Methods for determining the cut-off of the HALP score	Cut-off values of the HALP score	Median follow-up duration (months)	Outcomes	Variables adjusted
Cong 2017	China	RC	ESCC	39	60	100.0	II-IVa	Definitive chemoradiotherapy (docetaxel + cisplatin or carboplatin)	Median	48.3	27.2	OS, PFS	Age, tumor length, KPS, chemotherapy cycles, tumor location, TNM stage
Feng 2021	China	RC	ESCC	355	59	81.4	I-III	Radical resection (R0) ± adjuvant therapy	ROC curve analysis derived	31.8	34	OS	Age, BMI, TNM stage
Topal 2021	Turkey	RC	EC (Adenocarcinoma + SCC)	44	62	73.0	I-III	Curative resection	ROC curve analysis derived	43	20	OS	Age, sex, ASA class, BMI, tumor size, differentiation, tumor stage, location, and presence of anastomotic leak
Hu 2021	China	RC	ESCC	834	60	61.0	I-III	Radical esophagectomy (R0) ± adjuvant therapy	ROC curve analysis derived	38.8	52.8	OS	Age, tumor length, differentiation, invasion depth, lymph node metastasis, MVV, postoperative complications
Shi 2023	China	RC	ESCC	150	65	83.3	Unresectable locally advanced (Clinical Stages: II 16.7%, III 2.7%, IVa 80.6%)	CCRT	ROC curve analysis derived	23.1	27.5	OS, PFS	Age, sex, ZPS score, pre-radiotherapy recurrence/metastasis, recent efficacy (SD+PD vs CR+PR), and mGRIm score
Sucuoglu 2024	Turkey	RC	ESCC	45	55.8	42.2	Locally advanced (Stages not specified further; cT1-4, cN0/N+)	CRT with concurrent carboplatin/paclitaxel or cisplatin/5-FU	ROC curve analysis derived	42.4	20	OS, PFS	None
Xu 2024	China	RC	ESCC	190	61.4	81.1	Locally advanced (Stage II-IVa)	Neoadjuvant chemoimmunotherapy (paclitaxel-based chemo + PD-1 inhibitor) followed by radical esophagectomy	Optimal cut-off values determined using the survminer package in R	59	16	PFS	Age, sex, tumor stage, and operative blood loss
Yamamoto 2025	Japan	RC	ESCC	180	70	86.1	Clinical Stage 0–III (Resectable, curative intent)	Curative esophagectomy (± neoadjuvant chemotherapy/chemoradiotherapy)	NR	40	29.6	OS, PFS	Age, sex, tumor stage, lymph node metastasis, and lymph-vascular invasion
Hasegawa 2025	Japan	RC	ESCC	85	69	72.9	II-IV	Neoadjuvant chemotherapy (NAC: DCF/CF/CF+RT) followed by esophagectomy	Previous study defined	20	28.7	OS	None
Kharel 2025	Nepal	PC	Esophageal and esophagogastric junction cancers (Adenocarcinoma + SCC)	31	61.5	54.8	I-III	Curative-intent surgery (various types: McKeown, Transhiatal, etc.)	ROC curve analysis derived	38	1	OS	None

ASA, American Society of Anesthesiologists; BMI, body mass index; CCRT, concurrent chemoradiotherapy; CF, cisplatin plus 5-fluorouracil; CRT, chemoradiotherapy; DCF, docetaxel, cisplatin, and 5-fluorouracil; EC, esophageal cancer; ESCC, esophageal squamous cell carcinoma; FU, fluorouracil; GEC, gastroesophageal junction cancer; HALP, hemoglobin, albumin, lymphocyte, and platelet score; KPS, Karnofsky performance status; mGRIm, modified Gustave Roussy Immune score; MVV, maximal vital capacity; NAC, neoadjuvant chemotherapy; NR, not reported; OS, overall survival; PC, prospective cohort; PFS, progression-free survival; PR, partial response; RC, retrospective cohort; ROC, receiver operating characteristic; SD, stable disease; TNM, tumor-node-metastasis; ZPS, Zubrod performance status.

### Study quality evaluation

Study quality was assessed using the NOS. The detailed results are presented in **Table 2**. The NOS scores ranged from 6 to 9, indicating a moderate-to-high methodological quality overall. One study (17) achieved the maximum score of 9, reflecting strong cohort representativeness, adequate exposure ascertainment, comprehensive confounder adjustment, reliable outcome assessment, and sufficient follow-up. Five studies scored 8 (15, 16, 18, 19, 24), because these studies were primarily limited by insufficient and long follow-up durations, despite the appropriate control for age and other key confounders. One study scored 7, which was limited by the lack of representativeness of the exposed cohort and short follow-up duration (21). The other three studies scored 6, which were mainly constrained by lack of multivariable adjustment and limited follow-up. Overall, the quality assessment supports the internal validity of the included evidence, while highlighting variability in confounder control and follow-up adequacy, which was taken into account when interpreting the pooled results.

**Table 2:** Study quality evaluation *via* the Newcastle-Ottawa scale.

Study	Representativeness of the exposed cohort	Selection of the non-exposed cohort	Ascertainment of exposure	Outcome not present at baseline	Control for age	Control for other confounding factors	Assessment of outcome	Enough long follow-up duration	Adequacy of follow-up of cohorts	Total
Cong 2017	1	1	1	1	1	1	1	0	1	8
Feng 2021	1	1	1	1	1	1	1	0	1	8
Topal 2021	1	1	1	1	1	1	1	0	1	8
Hu 2021	1	1	1	1	1	1	1	1	1	9
Shi 2023	1	1	1	1	1	1	1	0	1	8
Sucuoglu 2024	1	1	1	1	0	0	1	0	1	6
Xu 2024	0	1	1	1	1	1	1	0	1	7
Yamamoto 2025	1	1	1	1	1	1	1	0	1	8
Hasegawa 2025	1	1	1	1	0	0	1	0	1	6
Kharel 2025	1	1	1	1	0	0	1	0	1	6

### Association between the HALP score and survival outcomes

**Figure 2:**
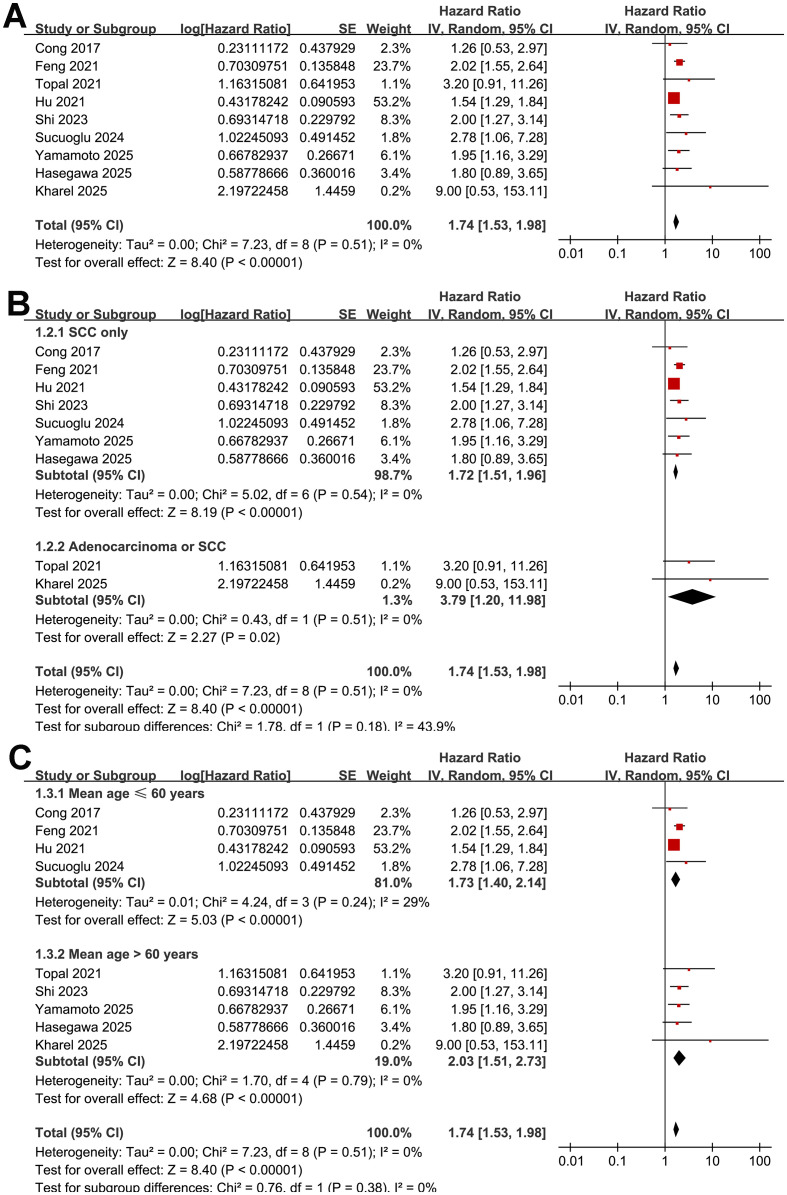
Forest plots for the meta-analysis of the association between the HALP score and OS of patients with EC or GEC. **(A)** The overall meta-analysis; **(B)** The subgroup analysis, according to the histology type of the cancer; **(C)** The subgroup analysis, according to the mean age of the patients.

**Figure 3:**
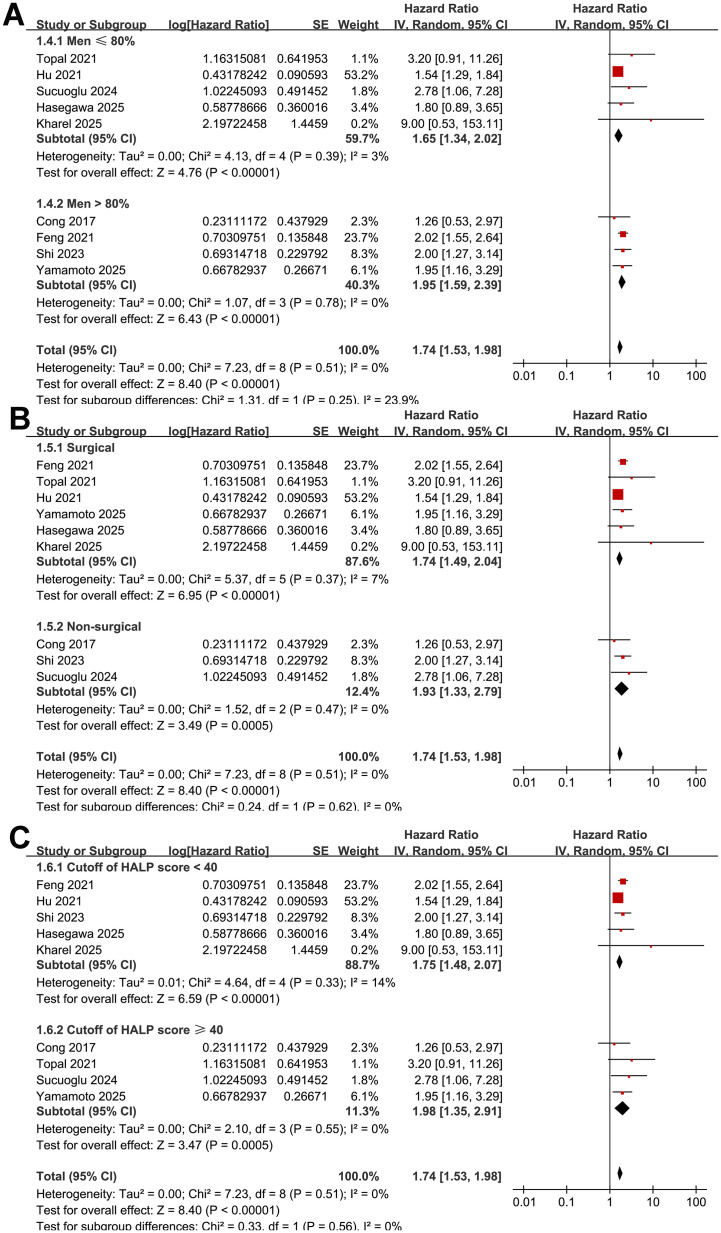
Forest plots for the subgroup analyses of the association between the HALP score and OS of patients with EC or GEC. **(A)** The subgroup analysis, according to the proportion of men; **(B)** The subgroup analysis, according to the main anticancer treatments; **(C)** The subgroup analysis, according to the cut-off value of the HALP score in each study.

**Figure 4:**
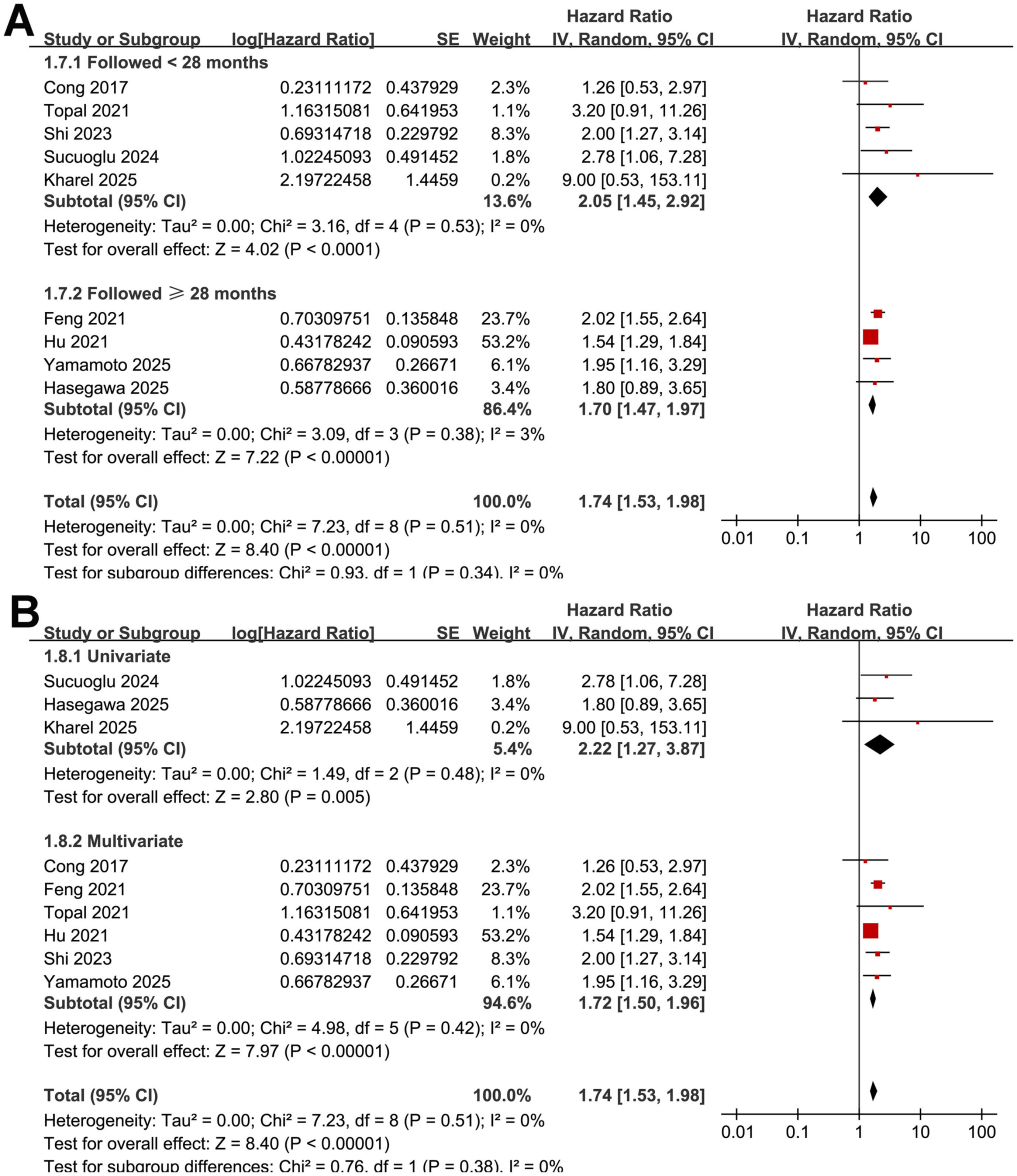
Forest plots for the subgroup analyses of the association between the HALP score and OS of patients with EC or GEC. **(A)** The subgroup analysis, according to the mean follow-up duration; **(B)** The subgroup analysis, according to the analytic model.

The pooled results of nine studies (15–20, 22–24) revealed that compared to patients with high HALP scores, EC or GEC patients with low HALP scores were associated with poor OS (HR: 1.74, 95% CI: 1.53-1.98, *p* < 0.001), without significant heterogeneity (*p* for Cochrane Q test = 0.51, *I^2^* = 0%; **Figure 2A**). By omitting one study at a time, the sensitivity analysis did not significantly change the results (HR: 1.66-2.00; all, *p* < 0.05). Additional sensitivity analyses limited to studies, in which HALP score cut-offs were determined using ROC curve analysis (16–20, 23), revealed similar results for OS (HR: 1.83, 95% CI: 1.51-2.23, *p* < 0.001; *I^2^* = 23%), supporting the robustness of the findings, despite the heterogeneity in cut-off derivation methods. Furthermore, the sensitivity analysis that excluded studies with NOS scores of ≥7 (15–17, 19, 24, 28) revealed similar results for OS (HR: 1.72, 95% CI: 1.50-1.96, *p* < 0.001; *I^2^* = 0%). The further subgroup analysis revealed similar results between studies that included patients with esophageal SCC only and patients with adenocarcinoma (HR: 1.72 *vs.* 3.79, *p* for subgroup difference = 0.18; **Figure 2B**), in studies with a mean patient age of ≤60 and >60 years old (HR: 1.73 *vs.* 2.03, *p* for subgroup difference = 0.38; **Figure 2C**), in patient cohorts where the proportion of men was ≤80% and >80% (HR: 1.65 *vs.* 1.95, *p* for subgroup difference = 0.25; **Figure 3A**), in studies where patients received surgical or non-surgical treatments (HR: 1.74 *vs.* 1.93, *p* for subgroup difference = 0.62; **Figure 3B**), and between studies with HALP score cut-off values of <40 and ≥40 (HR: 1.75 *vs.* 1.98, *p* for subgroup difference = 0.56; **Figure 3C**). Furthermore, consistent results were obtained from studies with a follow-up duration of <28 months and ≥28 months (HR: 2.05 *vs.* 1.70, *p* for subgroup difference = 0.34; **Figure 4A**), and studies with univariate and multivariate analyses (HR: 2.22 *vs.* 1.72, *p* for subgroup difference = 0.38; **Figure 4B**).

**Figure 5:**
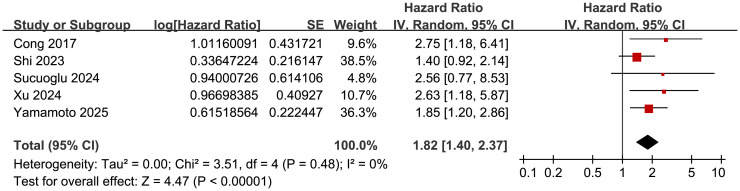
Forest plots for the meta-analysis of the association between the HALP score and PFS of patients with EC or GEC.

The further meta-analysis results for five studies (15, 19–21, 24) suggested that EC or GEC patients with low HALP scores were associated with poor PFS (HR: 1.82, 95% CI: 1.40-2.37, *p* < 0.001), without significant heterogeneity (*p* for Cochrane Q test = 0.48; *I^2^* = 0%; **Figure 5**). However, by excluding one study at a time, the sensitivity analysis did not significantly affect the results (HR: 1.74-2.15; all, *p* < 0.05). Consistently, additional sensitivity analyses limited to studies, in which HALP score cut-offs were determined using ROC curve analysis (19, 20), revealed similar results for PFS (HR: 1.50, 95% CI: 1.02-2.23, *p* = 0.04; *I^2^* = 0%). Furthermore, the sensitivity analysis that excluded studies with NOS scores of ≥7 (15, 19, 21, 24) revealed similar results for PFS (HR: 1.80, 95% CI: 1.36-2.39, *p* < 0.001; *I^2^* = 6%). Given that only five studies reported the PFS outcomes, these findings should be considered exploratory and interpreted with caution.

### Publication bias

**Figure 6:**
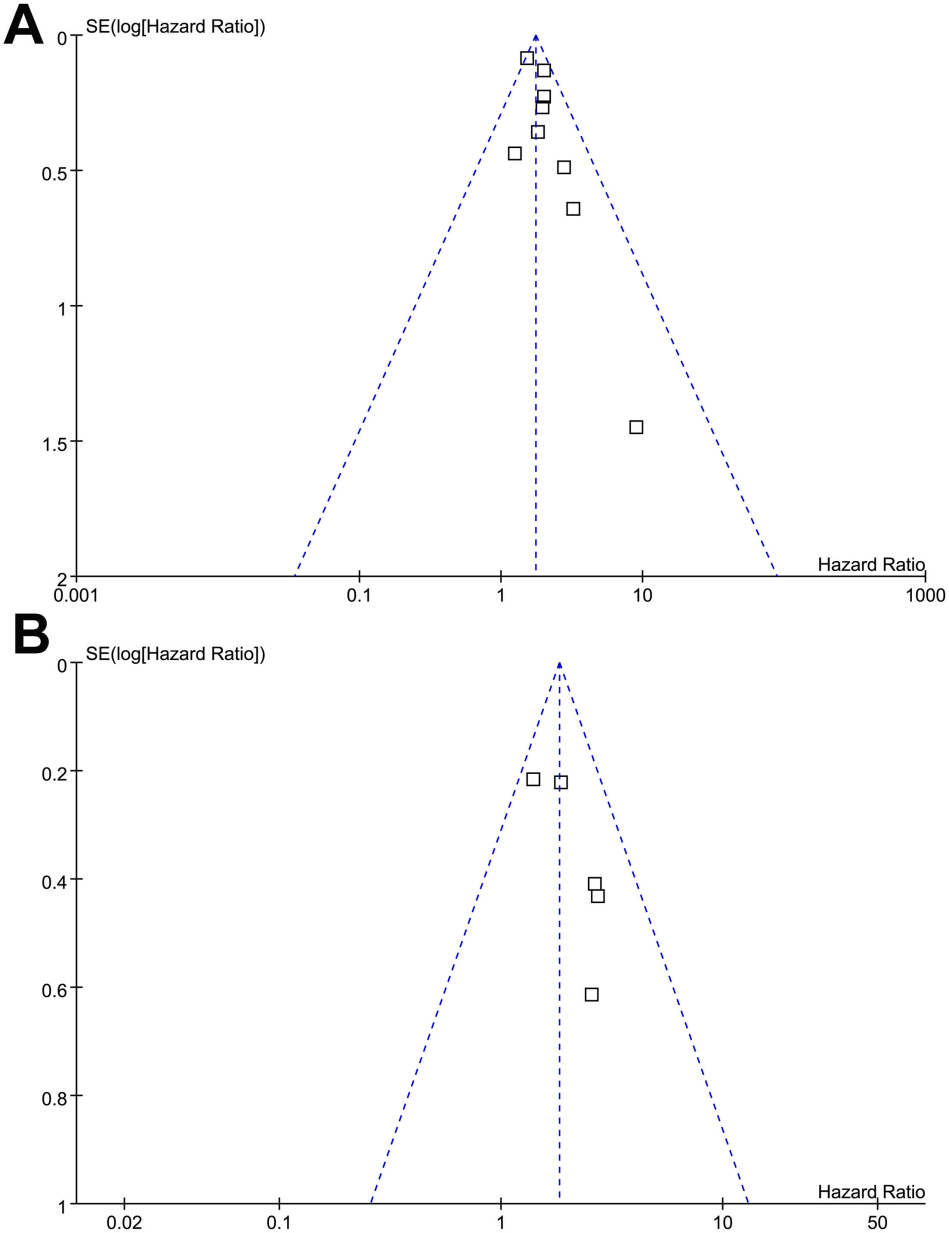
Funnel plots for estimating the potential publication bias underlying the meta-analyses, with the association between the HALP score and OS/PFS of patients with EC or GEC. **(A)** Funnel plots for the meta-analysis of the association between the HALP score and OS; **(B)** Funnel plots for the meta-analysis of the association between the HALP score and PFS.

**Figures 6A, B** present the funnel plots for evaluating the publication bias underlying the meta-analyses of the association between the HALP score and OS/PFS in patients with EC or GEC. The plots appeared to be symmetrical on visual inspection, but did not suggest a high risk of publication bias. For the primary outcome of OS, the Egger’s regression analysis did not suggest a high risk of publication bias (*p* = 0.41). For the secondary outcome of PFS, Egger’s regression analysis was not performed, because only five studies were included.

## Discussion

Across the available longitudinal evidence, the baseline HALP score emerged as the consistent prognostic indicator in patients with EC or GEC. In general, patients with low HALP scores experienced poorer survival outcomes, supporting the concept that host-related nutritional and inflammatory-immune status meaningfully contributes to prognosis beyond tumor-centered characteristics alone. Importantly, the direction of association was stable across studies, despite the differences in region, clinical stage distribution, treatment strategy, and HALP cut-off determination, suggesting that the HALP score captures a common biological vulnerability relevant to disease progression and survival in this population.

The observed prognostic signal was clinically plausible when the individual components of the HALP score were considered. Hemoglobin reflects the oxygen-carrying capacity, and can be a marker of chronic disease burden. Anemia may worsen tissue hypoxia, reduce functional reserve, and impair tolerance to surgery, chemoradiotherapy, and multimodal treatment, potentially increasing the likelihood of treatment interruptions and postoperative morbidity (29, 30). Albumin is a well-established surrogate of nutritional status (31) and systemic inflammation (32). Hypoalbuminemia often coexists with cancer cachexia, impairs wound healing, and has susceptibility to infection, which may translate into higher non-cancer mortality, as well as compromised delivery of anticancer therapy (33). Lymphocyte count serves as an accessible marker of antitumor immune competence (34). Lymphopenia may indicate impaired immune surveillance and weaker cytotoxic response, allowing for a more aggressive tumor behavior and earlier recurrence (35). Platelet count reflects inflammatory activation, and may contribute to tumor progression through the platelet-mediated shielding of circulating tumor cells, facilitation of adhesion/extravasation, adhesion/extravasation, and promotion of angiogenesis (33). Taken together, a low HALP score represents the convergence of anemia, malnutrition, immune suppression, and inflammation-driven thrombocytosis, which is an adverse host milieu that can reasonably contribute to both worse OS and shorter PFS.

The subgroup analyses further supported the robustness and potential generalizability of the findings. The association between low HALP scores and poor OS was directionally consistent in studies restricted to esophageal SCC, and in studies that included adenocarcinoma and/or gastro-esophageal junction tumors, suggesting that the HALP score may act as a host-prognostic marker across histologic subtypes, rather than being specific to one biology. Similarly, the consistent effects across the age strata and sex distributions indicate that the HALP score retains prognostic relevance in different demographic contexts, even though the baseline nutritional and inflammatory profiles can substantially vary by age and comorbidity burden. Furthermore, the stability of results across the surgical and non-surgical treatment subgroups implied that the HALP score may capture factors that influence both peri-treatment resilience (*e.g.* postoperative recovery and ability to complete the chemoradiotherapy) and longer-term oncologic control. Moreover, similar associations were observed across studies that used different HALP score cut-offs, which is reassuring from a practical perspective. Although the heterogeneity of the cut-off values may complicate clinical application, the consistent association suggests that the prognostic value of the HALP score is not driven by a single dichotomization threshold. The sensitivity analyses strengthened the confidence in the main conclusion. The pooled estimates did not materially change when individual studies were omitted, indicating that the overall inference was not driven by a single influential cohort or an outlier estimate. In addition, the absence of statistical heterogeneity in the main analyses suggests that within the limitations of study-level aggregation, the direction and magnitude of effect were relatively coherent across datasets. Nevertheless, the apparent homogeneity should be interpreted with caution, since the low *I^2^* did not exclude the clinically important between-study differences, particularly when studies shared similar designs, populations, or analytical approaches. In addition, although additional sensitivity analyses that excluded studies without ROC-derived cut-offs yielded similar results, supporting the robustness of the observed associations, the substantial variability in HALP cut-off values across studies remain an important limitation for clinical implementation. Different threshold derivation methods and patient populations may contribute to the inconsistent categorization of risk across studies. Future studies should further evaluate whether HALP is better modeled as a continuous variable, which may preserve more prognostic information and prevent arbitrary dichotomization, or determine whether standardized and clinically validated cut-off values can be established for specific treatment settings and patient populations.

The present work had several strengths. The literature search was up to date, and spanned multiple major databases, and merely longitudinal designs were included, improving the temporal relevance of exposure assessment to subsequent outcomes. Furthermore, the meta-analysis focused on time-to-event endpoints and synthesized HRs, which are appropriate for survival outcomes. In addition, the work incorporated multiple sensitivity and prespecified subgroup analyses to evaluate the robustness, and explore whether key clinical factors might modify the association. Furthermore, the study quality was systematically assessed using a validated tool. However, there were several limitations that also merit consideration. First, the evidence base was dominated by retrospective cohorts, which are susceptible to selection bias, incomplete data capture, and residual confounding. These features may inflate or attenuate the associations, and limit the causal interpretation (34). Second, although publication bias was not suggested for OS, the possibility cannot be fully excluded, particularly given the small number of studies for some outcomes and subgroups, and the limited power of asymmetry tests in meta-analyses with modest study counts. Third, clinical heterogeneity existed across studies in cancer stage distributions, treatment regimens (surgery, neoadjuvant/adjuvant strategies, definitive chemoradiotherapy, and emerging chemoimmunotherapy approaches), and definitions of follow-up and progression. These differences cannot be comprehensively evaluated without individual participant data (IPD). In addition, the present study was based on published aggregate data, which precluded the evaluation of whether the HALP score provides incremental prognostic value beyond conventional clinicopathological factors using metrics, such as C-index improvement, time-dependent AUC, net reclassification improvement, or decision curve analysis. Future prospective studies and IPD meta-analyses are needed to determine the additional prognostic utility and clinical applicability of HALP scores when integrated with established prognostic models. Fourth, the adjustment strategies varied across studies, and several cohorts only reported unadjusted estimates, raising the concern that some of the observed associations may reflect confounding by performance status, comorbidity, baseline disease burden, or other prognostic variables not uniformly controlled. Although several included studies had relatively lower NOS scores and lacked multivariable adjustment (20, 22, 23), the additional sensitivity analyses that excluded these studies yielded similar results, supporting the stability of the observed associations. Furthermore, the findings on PFS should be interpreted with caution, because only a limited number of studies were available for analysis, restricting the statistical power, and limiting the ability to formally assess publication bias. Another limitation is that the present meta-analysis focused primarily on baseline HALP scores, because the included studies generally did not provide sufficient data on dynamic changes in HALP during treatment. Longitudinal alterations in the HALP score before and after neoadjuvant therapy, chemoradiotherapy, or surgery may better reflect the treatment tolerance, nutritional decline, and evolving systemic inflammation, and can potentially provide additional prognostic information beyond a single baseline measurement. Future prospective studies are needed to evaluate the prognostic significance of dynamic HALP changes during treatment and follow-up. Finally, the HALP score was analyzed as a categorical exposure (high *vs.* low) based on study-specific cut-offs, which may reduce the information, and complicate direct clinical implementation. The optimal cut-off likely varied by population and treatment setting, and requires further validation.

### Clinical implications

Clinically, the HALP score is an attractive approach, because it is inexpensive, routinely available, and easily calculated (9), making it a pragmatic tool for baseline risk stratification in EC/GEC. If prospectively validated, the HALP score can help identify patients who may benefit from intensified nutritional support, prehabilitation, closer monitoring for complications, or tailored treatment intensity (35). Furthermore, the HALP score may be useful for counseling and shared decision-making. However, given the observational nature of the evidence and potential confounding, the HALP score should not be interpreted as a stand-alone determinant of prognosis or a trigger for altering oncologic management without supporting clinical context. Future research should prioritize prospective multicenter cohorts with standardized HALP measurement timing, harmonized cut-off derivation (or continuous modeling), and comprehensive adjustment for key prognostic factors. The IPD meta-analysis would be particularly valuable for assessing the dose-response relationships, exploring the interactions with stage and treatment modality, and defining clinically actionable thresholds. Interventional studies that evaluate whether correcting modifiable HALP score components (e.g. nutritional optimization, anemia management, and inflammation control, when appropriate) can improve treatment tolerance and outcomes are warranted. However, since the present meta-analysis was based on observational evidence, it could not be established whether the modification of HALP components directly improves prognosis. Therefore, prospective interventional studies are needed before therapeutic strategies that target HALP-related factors can be recommended in clinical practice.

## Conclusions

In conclusion, the present evidence indicates that a low baseline HALP score is consistently associated with worse survival outcomes in patients with EC or GEC. Although the findings support the HALP score as a promising, readily accessible prognostic biomarker, these should be interpreted with caution due to the predominance of retrospective data, heterogeneity in clinical contexts, and potential residual confounding. Further well-designed prospective studies are needed before the HALP score can be fully integrated into routine prognostic assessment and clinical decision pathways.

## Data Availability

The original contributions presented in the study are included in the article/supplementary material. Further inquiries can be directed to the corresponding author.
